# Corticosteroids in the Treatment of Pseudomembranous Colitis: A Report of 3 Cases

**DOI:** 10.4021/gr469w

**Published:** 2012-09-20

**Authors:** Ella Sykes, Patrick McDonald, Paul K. Flanagan

**Affiliations:** a Department of Gerontology, Royal Liverpool University Hospital, Liverpool, L7 8XP, UK; b Department of Medicine, Southport and Formby District General Hospital, Town Lane, Kew, Southport, PR8 6PN, UK; c Department of Gastroenterology, Royal Liverpool University Hospital, Liverpool, L7 8XP, UK

**Keywords:** Clostridium, Colitis, Pseudomembranous, Corticosteroids

## Abstract

*Clostridium difficile* associated diarrhoea has been an increasing problem in the last decade in the UK and is a cause of significant morbidity. At the most severe end of the spectrum it causes pseudomembranous colitis which has a significant associated mortality rate and can be refractory to standard treatments. Here we present three cases of proven pseudomembranous colitis in which systemic corticosteroids were used as an adjunct to treatment, raising the possibility of a new treatment option for this difficult condition.

## Introduction

*Clostridium difficile*, first linked to diarrhoea and colitis following antibiotic therapy in the 1970s, is an anaerobic bacterium found in low numbers in approximately 4% of the healthy adult population [1, 2]. *Clostridium difficile* associated diarrhoea (CDAD) is a major health burden and the UK saw a year on year increase in infection rates, and deaths, from *C. difficile* until 2006 [3]. Rates have fallen since 2007 but it remains a significant cause of morbidity and mortality was involved in 1.1% of all deaths between 2006 and 2010 [4].

Standard treatment (Table 1) includes withdrawal of precipitating medications and treatment with oral vancomycin or metronidazole [5]. Newer treatments include immunoglobulins, vaccination, novel antibiotics and probiotics. Corticosteroids are mentioned in some older textbooks as a treatment for CDAD but there is very little evidence to support this. We found one case report of a child with severe pseudomembranous colitis in which steroids were successfully used as an adjunct to established therapy [6]. Here we describe three cases of recurrent *Clostridium difficile* infection with endoscopically confirmed pseudomembranous colitis and their response to corticosteroid treatment.

**Table 1:** Classification and Treatment of *Clostridium difficile* Associated Diarrhoea [5]

Severity of Disease	Definition	Treatment
Mild	≤ 3 stools a day and a normal White cell count (WCC)	Oral metronidazole 400 - 500 mg tds for 10 - 14 days
Moderate	3-5 stools a day and raised WCC (< 15 × 10^9^/L)	Oral metronidazole 400 - 500 mg tds for 10 - 14 days
Severe	WCC > 15 × 10^9^/L, temperature > 38.5 °C, acute rising creatinine, or abdominal or radiological signs of acute colitis	Oral vancomycin 125 mg QDS 10 - 14 days. If not responding, high dose vancomycin (max 500 mg QDS) via NGT +/- IV metronidazole 500 mg TDS or IV immunoglobulins 400 mg/kg
Life threatening	Hypotension, partial or complete ileus, toxic megacolon or evidence of severe disease on computed tomography	Vancomycin 500 mg QDS 10 - 14 days via NGT or PR + IV metronidazole 500 mg QDS. Consider colectomy
First recurrence		Repeat same antibiotic as initial episode (If first treatment was with metronidazole and the recurrence is severe use vancomycin)
Subsequent recurrence		Oral vancomycin 125 mg QDS

## Case Report

### Case 1

A 54-year-old female presented with *Clostridium difficile* associated diarrhoea (CDAD) following a course of flucloxacillin in the community. On initial assessment she fell into the moderate category for CDAD (Table 1), and was managed with a 10 day course of oral metronidazole with good clinical response.

Ten days after completion of her initial treatment, she represented with watery diarrhoea and abdominal pain with a positive test for *C. difficile* stool toxin (CDT). Due to the lack of clinical response to treatment, high C reactive protein (CRP, 149 mg/L, normal < 5 mg/L) and continued diarrhoea, she proceeded to CT abdomen. This showed left colonic bowel wall thickening suggestive of colitis, placing her in the severe category for classification of CDAD (Table 1).

Despite treatment with vancomycin and metronidazole her clinical condition deteriorated by day 4, with development of pyrexia (38.5 °C), tachycardia (125 bpm) and increasing inflammatory markers (CRP 236). Flexible sigmoidoscopy confirmed pseudomembranous colitis both macroscopically and histologically. Metronidazole was discontinued and she was commenced on intravenous hydrocortisone 100 mg four times per day (qds) with oral vancomycin 125 mg qds. Within a further 2 days stool frequency had reduced to 3 times per day, she was apyrexial (36.4 °C), heart rate had normalised (64 bpm) and inflammatory markers were reducing (CRP 132). After nine days of intravenous hydrocortisone and oral vancomycin her bowel frequency was once a day, CRP was 15, the patient was apyrexial and heart rate was 59 bpm.

She was discharged on 30 mg prednisolone once daily followed by a slowly reducing regimen. Repeat endoscopy 1 month after initial sigmoidoscopy was normal and outpatient follow up at 5 months confirmed sustained clinical response.

### Case 2

Our second case was a 73 year old female with multiple admissions due to *Clostridium difficile* associated diarrhoea. She initially presented with moderate-severe CDAD (Table 1) following a course of flucloxacillin in the community and was successfully treated with a ten day course of Metronidazole 400 mg tds. She re-presented one week after discharge, with features of moderate CDAD, and was again successfully treated, this time with ten days of oral vancomycin 125 mg qds.

**Figure 1:**
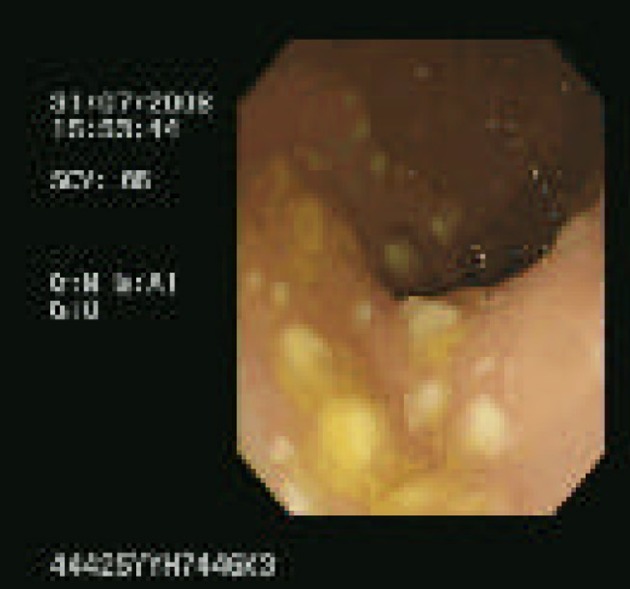
Endoscopy showing a classic pseudomembranous appearance, with multiple plaques from the rectum to the distal sigmoid.

Her third presentation was ten days after last discharge but on this occasion with pyrexia (38.6 °C), tachycardic (117 bpm) and raised inflammatory markers (CRP 87). She was prescribed oral vancomycin 125 mg qds but due to slow clinical response proceeded to flexible sigmoidoscopy on day 8. Endoscopy showed a classic pseudomembranous appearance, with multiple plaques from the rectum to the distal sigmoid (Fig. 1). In view of the findings corticosteroid treatment was added to her antibiotic therapy. At the time of commencing steroid treatment she was pyrexial (37.6 °C), tachycardic (114 bpm) and had raised inflammatory markers (CRP 112). Two days after commencing steroids her stool frequency normalised, she was apyrexial, heart rate was 90 bpm and inflammatory markers were reduced (CRP 6.6). She received 30 mg prednisolone for 7 days, followed by a slow reducing dose and completed a reducing course of vancomycin over 2 weeks. By day 14 of the final admission she had a complete clinical response and had no further relapses.

### Case 3

This 91-year-old female presented with moderate severity CDAD (Table 1) following a course of co-amoxiclav in the community and was commenced on oral metronidazole 400 mg tds. Despite ten days of Metronidazole her diarrhoea persisted although the patient was apyrexial (36.5 °C) with normal heart rate (58 bpm) and near normal inflammatory markers (CRP 11.2). She was then given a prolonged course of oral vancomycin 125 mg qds but her diarrhoea persisted so flexible sigmoidoscopy was performed. At the time of sigmoidoscopy the patient had no markers of severe disease although inflammatory markers were raised (CRP 25).

Flexible sigmoidoscopy confirmed pseudomembranous colitis so oral corticosteroids were commenced in addition to vancomycin treatment. Prednisolone 30 mg was given once daily for fourteen days with no gradual reduction, 125 mg Vancomycin qds was given initially, reduced to tds after one week, bd after another week and od for the last week. Within 3 days of starting corticosteroids her stool was formed and inflammatory markers had reduced (CRP 3.2). There were no subsequent episodes of CDAD.

## Discussion

We have presented three cases with typical histories for the development of *C. difficile* diarrhoea whose clinical course was complicated by pseudomembranous colitis. In all 3 cases corticosteroids were used as an adjunct to standard antibiotic therapy and whilst there is heterogeneity in both their clinical pictures, and the doses of steroids used, an improvement was seen following the addition of steroid therapy. The patients may have improved without steroids but in each case a significant clinical response was seen within 3 days of steroids starting. These cases highlight the importance of investigating for pseudomembranous colitis in patients not responding to treatment for *C. difficile* diarrhoea and raise the possibility of using steroids, in addition to antibiotics, for pseudomembranous colitis.

*Clostridium difficile* (*C. difficile*) is a spore forming, anaerobic, Gram positive bacillus which causes mucosal damage via the release of two toxins, A and B. Toxin A loosens epithelial cell junctions and is both enterotoxic and cytotoxic. Toxin B is a potent cytotoxin and causes breakdown of the cytoskeleton. As well as their cytotoxic effects the toxins induce cytokine production causing a marked inflammatory response [7].

Over the last 12 years, the incidence of CDAD has risen sharply and this has coincided with the recognition of a hypervirulent strain of *Clostridium difficile*. Ribotype 027 is the dominant hypervirulent strain and these strains result in more severe disease with higher relapse and mortality rates. This more severe pattern of disease is thought to be due to a combination of production of a binary toxin (in addition to toxin A and B), increased spore formation, increased adherance to human intestinal epithelial cells and resistance to fluroquinolones and erythromycin [8].

*C. difficile* can be carried asymptomatically or cause disease ranging from mild diarrhoea to fulminant colitis. Carriage rates in the general population are 3%, but as high as 20% in hospitalised patients [9]. There were over 50,000 cases in England in 2007 NOTEREF _Ref260308284 \h \* MERGEFORMAT and the U.S.A has seen a 23% annual increase in cases with a doubling of case fatality rates [5, 10]. Up to 25% of affected patients may develop pseudomembranous colitis and 1-3% progress to fulminant colitis [11, 12]. The overall case mortality of *C. difficile* associated disease has been reported as 2.2% but rises to 24% in fulminant colitis treated with colectomy, reaching 45% for those managed conservatively [5, 13].

Current treatment for established *C. difficile* diarrhoea follows the principals of supportive care, withdrawing offending antibiotics and Metronidazole or Vancomycin to eradicate *C. difficile* [5]. In most cases this will produce a quick response but some patients will have a more refractory course. To date research into newer treatments has focused on toxin binding (Anion resins, Immunoglobulins), improved host immune response (vaccination), alternative antibiotics (Rifaxamin, Nitazoxanide, OPT-80, Tinidazole) and altering gut flora (probiotics, faecal bacteriotherapy) [14]. These treatments may help reduce inflammation they do not target the process directly. Patients with pseudomembranous colitis have a marked inflammatory response in the colonic mucosa with some histological similarities (epithelial necrosis and neutrophil infiltration) to inflammatory bowel disease (IBD) where steroids are widely used.

Steroids were commonly used in the treatment of pseudomembranous colitis prior to the identification of *C. difficile* as the causative agent. A case series from 1976 found their use in up to 47% of cases with reported good efficacy [15, 16]. However, despite this early enthusiasm, modern use is limited, with only one contemporary case in the literature. Cavagnaro successfully used i.v. Methylprednisolone followed by oral Prednisolone, as an adjunct to standard therapy, in a 5-year-old with severe pseudomembranous colitis [6]. This is supported by experimental data where glucocorticoids modulate the inflammatory process in rats with *C. difficile* enteritis, whilst conversely a lack of endogenous steroid causes a more severe disease [17]. Steroids have multiple actions which include blocking cytokine production, reducing leucocyte accumulation and antagonising neutrophil adhesion and macrophage function [18]. *C. difficile* toxins promote cytokine release (IL-6, IL-8, IL-1β) and neutrophil recruitment leading to an inflammatory response and it is conceivable steroids may modulate this effect [19].

However a more recent study from 2010 found a significantly increased CDAD associated mortality in patients treated with concomitant glucocorticoids for other indications when compared to patients with CDAD not on glucocorticoids. Although in this series cause of mortality was sepsis in 18% of cases and not necessarily directly related to *Clostridium difficile* infection.

A counterpoint to this study is given by the literature relating to inflammatory bowel disease and *Clostridium* infection. Enteric infection (including *C. difficile*) can be found as the precipitant of flares of IBD in around 10% of cases [20]. In these patients, treatment with corticosteroids is not associated with a worse clinical outcome, suggesting they may also be safe in the wider context of CDAD [21].

Whilst this small case series cannot form the basis for a change in practice it suggests there may be a larger role for the use of steroids in pseudomembranous colitis and further studies may be warranted to look at this in more detail.

## References

[1] Bartlett JG, Chang TW, Gurwith M, Gorbach SL, Onderdonk AB (1978). Antibiotic-associated pseudomembranous colitis due to toxin-producing clostridia. N Engl J Med.

[2] Miyajima F, Roberts P, Swale A, Price V, Jones M, Horan M, Beeching N (2011). Characterisation and carriage ratio of Clostridium difficile strains isolated from a community-dwelling elderly population in the United Kingdom. PLoS One.

[3] Mooney H (2007). Annual incidence of MRSA falls in England, but C difficile continues to rise. BMJ.

[4] Office for National Statistics Deaths involving Clostridium difficile: England and Wales, 2006 to 20102011.

[5] Department of Health Clostridium difficile infection: How to deal with the problem22008.

[6] Cavagnaro C, Berezin S, Medow MS (2003). Corticosteroid treatment of severe, non-responsive Clostridium difficile induced colitis. Arch Dis Child.

[7] Hookman P, Barkin JS (2009). Clostridium difficile associated infection, diarrhea and colitis. World J Gastroenterol.

[8] Cartman ST, Heap JT, Kuehne SA, Cockayne A, Minton NP (2010). The emergence of 'hypervirulence' in Clostridium difficile. Int J Med Microbiol.

[9] Hurley BW, Nguyen CC (2002). The spectrum of pseudomembranous enterocolitis and antibiotic-associated diarrhea. Arch Intern Med.

[10] Zilberberg MD, Shorr AF, Kollef MH (2008). Increase in adult Clostridium difficile-related hospitalizations and case-fatality rate, United States, 2000-2005. Emerg Infect Dis.

[11] Kelly CP, LaMont JT (1998). Clostridium difficile infection. Annu Rev Med.

[12] Longo WE, Mazuski JE, Virgo KS, Lee P, Bahadursingh AN, Johnson FE (2004). Outcome after colectomy for Clostridium difficile colitis. Dis Colon Rectum.

[13] Sailhamer EA, Carson K, Chang Y, Zacharias N, Spaniolas K, Tabbara M, Alam HB (2009). Fulminant Clostridium difficile colitis: patterns of care and predictors of mortality. Arch Surg.

[14] Kelly CP, LaMont JT (2008). Clostridium difficile—more difficult than ever. N Engl J Med.

[15] Goodman MJ, Truelove SC (1976). Intensive intravenous regimen for membranous colitis. Br Med J.

[16] Keeffe EB, Katon RM, Chan TT, Melnyk CS, Benson JA (1974). Pseudomembranous enterocolitis. Resurgence related to newer antibiotic therapy. West J Med.

[17] Castagliuolo I, Karalis K, Valenick L, Pasha A, Nikulasson S, Wlk M, Pothoulakis C (2001). Endogenous corticosteroids modulate Clostridium difficile toxin A-induced enteritis in rats. Am J Physiol Gastrointest Liver Physiol.

[18] Boumpas DT, Chrousos GP, Wilder RL, Cupps TR, Balow JE (1993). Glucocorticoid therapy for immune-mediated diseases: basic and clinical correlates. Ann Intern Med.

[19] Voth DE, Ballard JD (2005). Clostridium difficile toxins: mechanism of action and role in disease. Clin Microbiol Rev.

[20] Navarro-Llavat M, Domenech E, Bernal I, Sanchez-Delgado J, Manterola JM, Garcia-Planella E, Manosa M (2009). Prospective, observational, cross-sectional study of intestinal infections among acutely active inflammatory bowel disease patients. Digestion.

[21] Ananthakrishnan AN, Guzman-Perez R, Gainer V, Cai T, Churchill S, Kohane I, Plenge RM (2012). Predictors of severe outcomes associated with Clostridium difficile infection in patients with inflammatory bowel disease. Aliment Pharmacol Ther.

